# The effect of biopolymer gel derived from sugarcane on healing of traumatic oral ulcers: an experimental study

**DOI:** 10.1590/acb390724

**Published:** 2024-03-11

**Authors:** Lívia Mirelle Barbosa, Lívia Maria Lopes de Oliveira, Camylla Pinheiro Verissimo Queiroz, Bruna Andrade Santos Freitas, Tarciana Maria Pereira de Lima, José Lamartine de Andrade Aguiar, Martinho Dinoá Medeiros, Danyel Elias da Cruz Perez, Gustavo Pina Godoy

**Affiliations:** 1 Universidade Federal de Pernambuco – Recife (PE) – Brazil.

**Keywords:** Oral Ulcer, Re-Epithelialization, Cellulose

## Abstract

**Purpose:**

The extracellular polysaccharide (EPS) is produced by the bacterium *Zoogloea* sp. and plays a positive role in tissue repair. The purpose of this study was to clinically and histologically compare the effects of EPS in the healing of traumatic oral ulcers in rats with the effects of triamcinolone.

**Methods:**

Ulcers were induced in the oral mucous of 45 male Wistar rats, divided into three groups: control group, treated with triamcinolone, and treated with biopolymer gel. In the clinical evaluation, we considered the weight variation of the animals and the size of the lesion area, at baseline and on treatment days 1, 3 and 7. The histological parameters evaluated were the type and intensity of the inflammatory infiltration, the presence of necrosis and foreign body granuloma and the degree of re-epithelialization of the lesion.

**Results:**

The reduction of the lesion area was greater in the animals treated with EPS, with no difference in the intensity of the inflammatory infiltration between the groups on days 3 and 7 of treatment.

**Conclusions:**

The results suggest that topical application of EPS in traumatic oral ulcers of rats promotes faster repair than triamcinolone ointment, without increasing the intensity of inflammatory infiltration under the lesion.

## Introduction

Oral ulcerative lesions are common in dental practice^1^, but the large number of illnesses leading to this condition sometimes hampers their diagnosis^2^. Oral traumatic ulcers (TUs) can result from physical or chemical aggression to the oral mucosa^3^.

The most effective treatment for these lesions has not been fully established; several protocols using topical or systemic drugs which aim to relieve symptoms and accelerate repair of the region to allow reconstitution of the injured tissue have been reported^4^^,^ 5. The prescription of triamcinolone ointment in orabase is common for TU treatment^6^.

The use of herbal medicines in wound healing has been widely studied, since they have active ingredients such as alkaloids, triterpenes and biomolecules that interfere in one or more phases of repair^7^^,^ 8. Plants can be also an indirect source of extracellular polysaccharide-producing microorganisms which have applications in various industries^9^.

*Zoogloea* sp., a bacterial product from extracellular polysaccharides (EPS) (zooglan), was isolated from sugarcane molasses (*Saccharum officinarum*) in the agroindustrial environment of Northeast Brazil (Experimental Sugarcane Station). The resulting biopolymer, with physical and chemical characteristics suitable for the preparation of materials such as gels, membranes, sponges, granules, and prostheses, has been extensively studied for application in the medical industry^10^. During the experiments, no clinical signs of infection, allergies, intoxication, or tissue extrusion were observed^7^^,^ 11 ^,^ 12.

The application of biopolymer in granular form was studied in bone defects of the skullcap of rats. A membrane of the same material was biocompatible for filling bone cavities, avoiding deformity and providing bone repair^13^^,^ 14. Teixeira et al.^15^ evaluated the action of sugarcane biopolymer sponge film in the repair of oral ulcers provoked in rabbits. They observed that the film remained in the ulcer bed, functioning as a mechanical barrier. The complete healing time of lesions that received the film did not differ from those that did not.

In view of the positive results found in the experimental studies involving this biopolymer, especially in tissue repair in different organic structures, the objective of this study was to evaluate the action of the sugarcane biopolymer gel, clinically and histologically, in the healing of TUs.

## Methods

### Sample

The present study was submitted to the Ethics Committee on Animal Use, at the Universidade Federal de Pernambuco (protocol 0010/2018), and approved in accordance with National Institutes of Health guide for the care and use of laboratory animals (NIH Publications No.8023, revised 1978). Forty-five male rats (breed: *Rattus norvegicus*, family: Wistar variety Muridae) weighing 200–300 g were housed under controlled temperature and lighting conditions (21–23°C, 12 hours of light/12 hours of dark) so that there would be no increase in stress levels; the supply of food and filtered water was unlimited.

The animals were randomly divided by lot into three groups of 15 animals each:

- Group 1: negative control (no treatment);
- Group 2: positive control (treated with triamcinolone ointment in orabase);
- Group 3: experimental (treated with EPS gel, Universidade Federal de Pernambuco / PI0301912-8 A2).
  The groups were subdivided according to the analysis time–1, 3 and 7 days:
- Group 1: D1;
- Group 2: D1;
- Group 3: D1;
- Group 1: D3;
- Group 2: D3;
- Group 3: D3;
- Group 1: D7;
- Group 2: D7;
- Group 3: D7.

### Each group contained five animals.

#### Induction of oral mucosa ulcer and treatment protocol

The animals were submitted to general anesthesia by intramuscular injection of xylazine chloride (Rompum, Bayer do Brasil, São Paulo, Brazil, 10 mg / kg body weight) and Ketamine (Ketalar, Parke-Davis, Aché Laboratories, São Paulo, Brazil, 75 mg/kg body weight). After oral antisepsis with chlorhexidine 0.12% and installation of operative fields, an infiltrative anesthesia of 2% lidocaine was superficially performed in the inferior lip of the animal, promoting hydrodissection between the epithelium of the mucosa and the connective tissue. The same operator (LM) as previously described^16^^,^ 17 induced an ulceration of 5 mm in diameter, using a metal punch.

After the induction of the ulcer, the drugs in groups 2 and 3 were applied in such a way that the wounds were completely covered. During the days that followed, the drug was applied once per day in the morning under general anesthesia, ensuring the maintenance and action of the drug in the ulcerated area for at least two hours. Five rats from each group were euthanized using a dose of 150 mg / kg of the general anesthetic sodium pentobarbital (Tiopental) injected intraperitoneally, one, three and seven days after the surgical procedure.

#### Clinical evaluation

The animals were monitored daily. Their weight was documented on the day of injury induction (day 0) and on the day of euthanasia, as a means of indirectly evaluating the presence of pain in association with intraoral injury^16^. The clinical analysis of healing progress was done by comparing the size of the lesion at day 0 and on the day of euthanasia. The largest and smallest diameters of the ulcers were measured with a digital caliper, and the calculation of the ulcerated area (A) was performed following Cavalcante et al.’s methodology^16^:

- R = larger radius;
- r = smaller radius (A = π • r • R).

### Histological evaluation

The oral pathologist (GP) responsible for the evaluation of the slides was unaware of which treatment each animal had received. The criteria established as cellular events related to the healing process were: type and intensity of inflammatory infiltration, presence or absence of foreign body granuloma, presence or absence of necrosis, and the degree of tissue re-epithelialization, following the methods adapted from Peixoto et al.^18^ and Sinha and Gallagher^19^.

Specimens whose inflammatory infiltrations were restricted to one-third of the microscopic field were classified as grade I (slight infiltration); lesions with inflammatory cells present between one- and two-thirds of the microscopic field were defined as grade II (moderate infiltration); and lesions that exhibited inflammatory infiltration greater than two-thirds of the microscopic field were categorized as grade III (intense infiltration). The classification of the degree of re-epithelialization followed the scores:

- Grade 1: re-epithelialization of less than half the wound;
- Grade 2: re-epithelialization covering at least half of the wound;
- Grade 3: re-epithelialization covering the entire wound.

### Statistical analysis

Data were entered into an Excel worksheet, and the program IBM Statistical Package for the Social Sciences version 23 was used to obtain statistical calculations. Either F (analysis of variance) or Kruskal-Wallis’ tests were used for comparison between groups or between the times of the euthanasia of the animals, and either the paired Student’s t-test or the paired Wilcoxon’s test were used to compare initial and final evaluations. Pearson’s χ^2^ test was used for the comparison between the groups in relation to categorical variables, and Fisher’s exact test was used when conditions for the χ^2^ test could not be verified.

## Results

### Clinical analysis

Table 1 shows the statistical analysis of the weight variation of different animal treatment groups throughout the research. The absolute mean and percentage changes were all positive, indicating a mean weight loss in all groups throughout the study, with group 1 showing the highest loss and group 3 showing the lowest one. A statistically significant difference in absolute weight variation was observed between groups 1 and 3 and between groups 1 and 2. There was no statistically significant difference in weight variation between groups 2 and 3.

**Table 1:** Statistical analysis of animal weight variation (g) throughout the research of differing treatment groups.

	Group 1 (negative control group)	Group 2 (positive control group)	Group 3 (experimental group)
Evaluation	Average ± SD	Average ± SD	Average ± SD
	Median (P25, P75)	Median (P25, P75)	Median (P25, P75)
Beginning	308.33 ± 86.60^A^	238.00 ± 31.95^B^	253.07 ± 22.12^A^
	322.00 (220.00; 370.00)	233.00 (207.00; 253.00)	254.00 (239.00; 267.00)
End	279.73 ± 88.71^A^	222.53 ± 25.78^B^	248.00 ± 24.96^A^
	309.00 (167.00; 368.00)	219.00 (200.00; 234.00)	247.00 (229.00; 262.00)
Mean Differences			
Absolute (I – F)	28.60^A^	15.47^A^ ^B^	5.07^B^
Percentage	10.00	6.23	1.84
P-value	p^C^ = 0.006*	p^C^ < 0.001*	p^C^ = 0.262

SD: standard deviation;

* Significant difference of 5%;

A Kruskal-Wallis’ test with comparisons with the aforementioned test;

B F (analysis of variance) test with comparisons to the Tukey’s test;

C Paired Student’s t-test;

Source: Elaborated by the authors.

**Figure 1:**
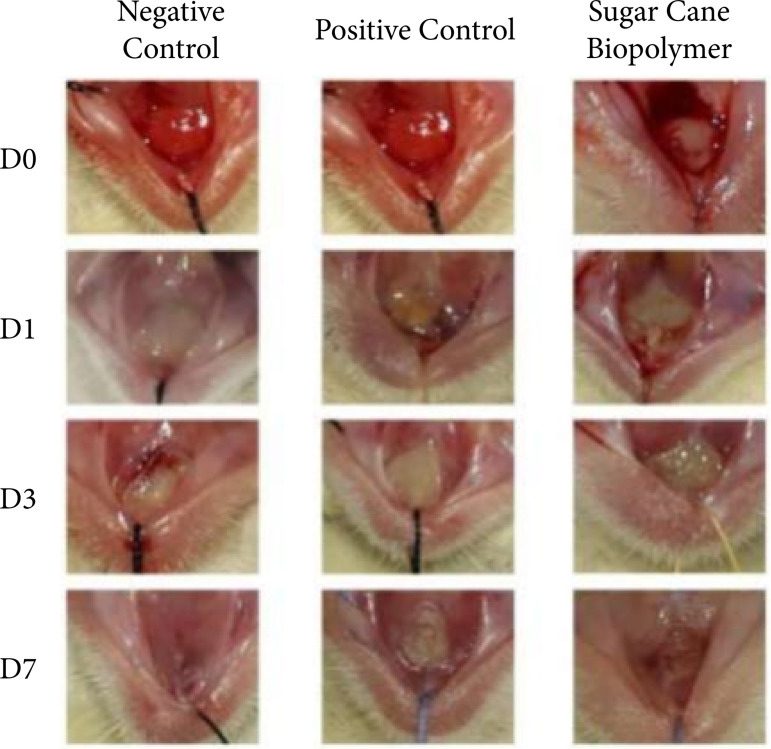
Clinical evolution of ulcers in the different groups.

Source: Elaborated by the authors.

After induction of the lesion (D1), it was already possible to observe the presence of ulceration covered by a fibrin layer of whitish-yellow membrane. Figure 1 shows the clinical evolution of ulcers in the different groups. Throughout the research, only two animals of the experimental group and three animals of the negative control group had complete repair of the lesion observed on D7. Table 2 shows the statistical analysis of the area of the lesions on the day of euthanasia (final area) compared to the initial area of the lesion, according to the treatment groups, with reduction in the three groups, during the three evaluation intervals (p < 0.05). When comparing lesion regression between groups, the only significant difference (p < 0.05) occurred on D7. Group 2 presented a mean area greater than groups 1 and 3 on D7, with no difference in the other groups.

**Table 2:** Statistical analysis of the lesion area (mm^2^) according to the day of euthanasia and treatment group^$^.

Days of evaluation	Group 1 Negative control	Group 2 Positive control	Group 3 experimental	P-value
	Average ± SD	Average ± SD	Average ± SD	
	Median (P25, P75)	Median (P25, P75)	Median (P25, P75)	
Day 1	16.80 ± 2.95*	16.37 ± 2.41*	15.99 ± 2.67*	p! = 0.925
	17.50 (13.81; 19.45)	15.62 (14.20; 18.92)	15.73 (13.47; 18.63)	
Average absolute difference (I – F)	2.83	3.26	3,65	p! = 0.925
Average percentage difference (I – F)	14.42	16.62	18.58	p! = 0.925
P-value	p^@^ = 0.080	p^@^ = 0.043*	p^@^ = 0.043*	
3 days	11.70 ± 2.03*	9.62 ± 2.87*	10.47 ± 2.92*	p! = 0.512
	11.40 (10.00; 13.55)	10.24 (7.15; 11.79)	10.64 (7.55; 13.31)	
Average absolute difference (I – F)	7.93	10.01	9,16	p! = 0.512
Average percentage difference (I – F)	40.42	50.99	46.67	p! = 0.512
P-value	p^@^ = 0.042*	p^@^ = 0.043*	p^@^ = 0.043*	
7 days	1.84 ± 2.47*	4.79 ± 0.41*	1.85 ± 1.21*	p! = 0.049*
	1.38 (0.00; 3.92)	4.79 (4.47; 5.11)	1.33 (0.82; 3.13)	
Average absolute differences (I – F)	17.79*	14.84*	17.78*	p! = 0.049*
Average percentage differences (I – F)	90.61*	75.60*	90.60*	p! = 0.049*
P-value	p^@^ = 0.042*	p^@^ = 0.042*	p^@^ = 0.043*	
P-value	p^#^ = 0.003*	p^#^ = 0.002*	p^#^ = 0.003*	

SD: standard deviation;

* significant difference of 5%;

! Kruskal-Wallis’ test for comparison between groups in each evaluation period with the aforementioned test;

@ Wilcoxon’s test pared between before and during each evaluation period for each group;

# Kruskal-Wallis’ test to make comparisons between times of evaluation in each group with comparisons with the aforementioned test;

Source: Elaborated by the authors.

### Histological analysis

**Figure 2:**
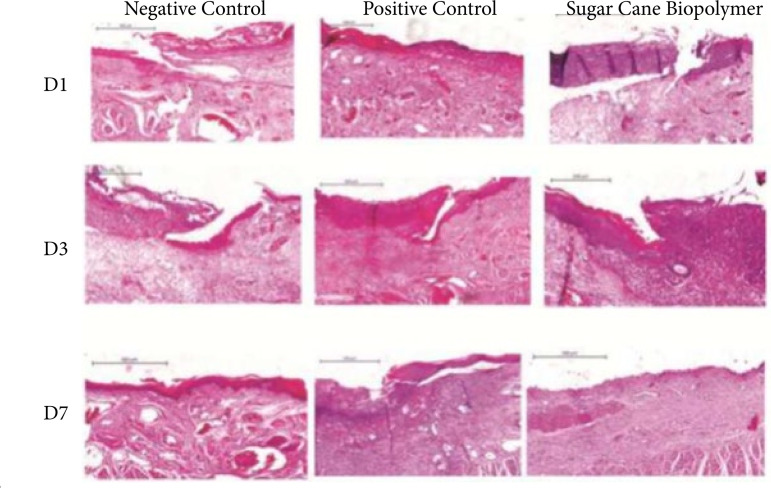
Histological analysis of the re-epithelialization process of traumatic ulcer in the three groups of euthanasia. **D1:** Presence of fibrin clot in the ulcerated area. **D3:** Initial formation of epithelium at the edges of the lesion. **D7:** Re-epithelialization throughout the lesion only in groups 1 and 3.

Source: Elaborated by the authors.

The results of the histological analysis of inflammatory infiltration are presented in Figs. 2a and 2b and Table 3. The presence of acute inflammatory infiltration was observed in all the animals euthanized on D1, whereas on D3 and D7 chronic inflammatory infiltration predominated. There was no statistically significant difference in the type of inflammatory infiltration between the groups in the evaluated periods.

**Table 3:** Histological analysis of variables: type of inflammatory infiltration, intensity of inflammatory infiltration, during evaluation days and groups.

Evaluation days	Variables	Group					P-value
		Group 1	Group 2	Group 3	Group total		
		(n = 5)	(n = 5)	(n = 5)	(n = 15)		
	Type of inflammatory infiltration						**
Day 1	Acute	5	5	5		15	
	Chronic	-	-	-		-	
	Type of inflammatory infiltration						p^^!^^ = 1.000
Day 3	Acute	1	-	-		1	
	Chronic	4	5	5		14	
	Type of inflammatory infiltration						p^!^ = 1.000
Day 7	Acute	1	-	-		1	
	Chronic	4	5	5		14	
Day 1	Intensity of inflammatory infiltration						p^!^ = 0.004*
	I	3	-	-		3	
	II	-	5	2		7	
	III	2	-	3		6	
Day 3	Intensity of inflammatory infiltration						p^!^ = 0.190
	I	-	2	-		2	
	II	2	-	3		5	
	III	3	3	2		8	
Day 7	Intensity of inflammatory infiltration						p^!^ = 0.134
	I	1	1	4		6	
	II	2	4	1		7	
	III	2	-	-		2	

* Significant difference of 5%;

undetermined due to insufficient responses in a singular category; !exact Fisher’s test; group 1: negative control; group 2: positive control; group 3: experimental. Source: Elaborated by the authors.

The analysis of the intensity of inflammatory infiltration revealed that only on D1 there was a statistically significant difference between the groups, in which the positive control group presented lower intensity of inflammatory infiltration. There was no necrosis or foreign body granuloma observed in any group.

**Figure 3:**
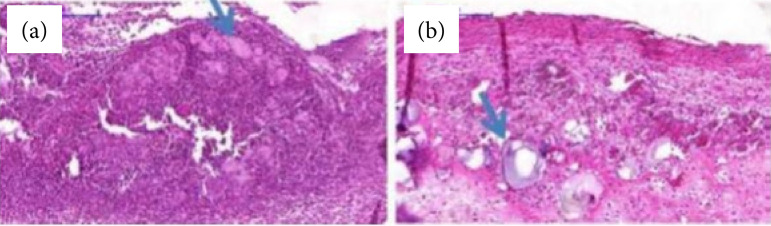
Histological evaluation of the ulcerated area on the first day after induction in animals of **(a)** the experimental groups and **(b)** the positive control groups. The presence of **(a)** extracellular polysaccharide (EPS) gel and triamcinolone ointment in orabase between the ulceration area and the fibrin membrane is observed.

Source: Elaborated by the authors.

On the first day (D1) after injury induction, the degree of re-epithelialization was similar between the groups (p > 0.05). On D3, re-epithelialization covered at least half of the wound in all animals of group 1 and 3, whereas in all animals of group 2 re-epithelialization covered less than half of the wound (p < 0.05). On D7, three of the five animals in groups 1 and 3 presented complete re-epithelialization of the wound, whereas no animals in group 2 presented complete re-epithelialization of the lesion (p = 0.09). These results can be observed in Fig. 3 and Table 4.

**Table 4:** Histological analysis of the degree of re-epithelialization of ulcers by evaluation days and treatment groups@.

Evaluationdays	Degree ofre-epithelialization	Group					P-value
		Group 1	Group 2	Group 3	Group total		
		(n = 5)	(n = 5)	(n = 5)	(n = 15)		
Day 1	1	5	5	5		15	p! = 1.000
	2	-	-	-		-	
	3	-	-	-		-	
		(A)	(B)	(A)			
Day 3	1	-	5	-		5	p! = 0.001*
	2	5	-	5		10	
	3	-	-	-		-	
Day 7	1	-	-	-		-	p! = 0.097
	2	2	5	2		9	
	3	3	-	3		6	

* Significant difference of 5%;

! Kruskal-Wallis’ test with comparisons with aforementioned test;

@ if the letters in parenthesis are different, there are significant differences between the corresponding groups;

group 1: negative control; group 2: positive control; group 3: sugar cane biopolymer; Source: Elaborated by the authors.

## Discussion

Various synthetic and phytotherapeutic products have been used in experimental studies for the treatment of TUs induced in animals^20^^,^ 21. In this study, a traumatic injury was induced in the lower lip of rats, to facilitate manipulation of the labial region without having to force open the animal’s mouth, which could interfere with the repair process. This locale, in addition, allowed for longer contact with orabase and gel in the area of the induced lesion.

Other studies have performed the induction of ulcers on the lower lip, produced chemically with either acetic acid and formocresol, or heated instruments^5^^,^ 17 ^,^ 22. In this study, we opted for surgical, non-chemical induction to mimic common traumatic ulcers in the oral cavity of humans, usually caused by maladaptive prostheses, dental crowns, fractured restorations, or accidental biting of the lip during chewing^16^. Another important factor in the choice of surgical induction was the standardization of wound size: all were made with a punch.

The weight variation of the animals was analyzed throughout the research, and it was observed that there was weight loss in all groups of animals, with the highest loss in group 1, compared to the other groups (p < 0, 05). It was inferred, therefore, that the animals which did not receive any medication had the greatest weight loss, which may be related to greater alimentary difficulties due to pain caused by untreated oral lesions. According to previous studies^8^^,^ 15 ^,^ 16 ^,^ 21, monitoring the weight of animals submitted to TU induction is an indirect means of evaluating the pain of animals.

An evaluation of TU treatment in rabbits using an EPS sponge film^15^ showed no significant weight variation between experimental and negative control animals. On the other hand, the application of the biopolymer gel used in this study directly at the injury site resulted in reduction of physical effects caused by food and chemical products, producing greater comfort for the rats, and resulting in less weight loss in comparison with the animals of the negative control group. It can be also hypothesized that the polymer composition of the gel with high purity of sugars (glucose 87.6%, xylose 8.6%, mannose 0.8%, ribose 1.7%, galactose 0.1%, arabinose 0.4%, and glucuronic acid 0.8%) promoted analgesic action at the site of the lesion, producing greater comfort and resulting in lower average weight loss in this group. The analgesic action of this gel may result from the reduction of local edema, since sugar is known to reduce edema and improve local circulation in wounds^23^^,^ 24.

On D7, group 2 presented a greater mean area of lesions than groups 1 and 3. This infers that the group treated with sugarcane biopolymer presented greater reduction of lesion size compared to animals treated with triamcinolone ointment in orabase.

Studies evaluating TU treatment in rats using aloe vera extract and copaiba oil, as opposed to topical corticosteroids, also found a higher mean area of ulceration in animals treated with topical corticosteroids, which concurs with the present study^7^^,^ 25. These results allow us to infer that topical use of corticosteroids in traumatic ulcers in oral mucosa can delay tissue repair, despite greater comfort for the animals. This finding may be explained by the modulatory action of the inflammatory process provoked by the drug. Since tissue repair is linked to the inflammatory process, the suppression of this process implies the delay of tissue repair^26^^,^ 27.

This study found no statistically significant mean differences between the negative and experimental control groups. This finding was also observed in studies that evaluated TU treatment in rats using extracts of aloe vera, *açaí*, chamomile, and copaiba^8^^,^ 25 ^,^ 28 ^,^ 29.

As a result of the histological analysis, it was possible to observe the presence of acute inflammatory infiltration in all animals sacrificed on the first day after the induction of the lesion; on the third and seventh day, the groups demonstrated a predominance of the chronic inflammatory infiltration. The differing intervals between the groups being euthanized demonstrated no difference between the groups in the type of inflammatory infiltration. These findings are compatible with the normal tissue repair process, especially when characterized by the absence of infection, thus verifying the anticipated evolution of the inflammatory process in the absence of infection^30^^,^ 31.

The use of EPS gel did not provoke an increase in the intensity of inflammatory infiltration in evaluated animals, diverging from results found in similar studies. It was expected that the inflammatory infiltration would be higher in the experimental group, since EPS releases a quantity of sugar capable of causing hyperosmolarity, which is considered to be a tissue irritant^7^^,^ 32 ^,^ 33. Hyperosmolarity is known to be a positive factor for the repair process, since it stimulates the formation of granulation tissue^13^^,^ 15 ^,^ 34. Since sugarcane gel did not cause intense inflammatory infiltration in the present study, it is possible to infer that this substance has potential to moderate inflammation.

The absence of necrosis and foreign body granuloma in the studied animals corroborates with studies involving cellulosic polysaccharide of sugarcane, confirming their biocompatibility. These findings allow us to infer that this material could be used in human research^7^^,^ 13 ^,^ 15 ^,^ 32 ^–^ 34.

In this study, it was possible to microscopically evaluate the presence of triamcinolone ointment and EPS gel between the ulcer and the fibrin membrane formed on the lesion. This finding is of great relevance, because, unlike triamcinolone ointment, EPS gel does not contain an orabase carrier, which acts as an adhesive and provides a protective covering, which facilitates extended contact of triamcinolone with oral tissues. Similarity of chemical composition explains this finding, since both materials present cellulose as a component^10^^,^ 35.

The histological evaluation showed that the degree of oral ulcer re-epithelialization of the animals was higher between groups 1 and 3 on the third and seventh day of evaluation, with a statistically significant difference on D3. Such histological findings are compatible with the clinical findings of the lesion area, demonstrating the importance of clinical and histological correlation in experimental studies.

The best results of the degree of re-epithelialization between the experimental and negative control groups highlight the delayed action of topical corticosteroids in oral ulcer tissue repair. Unlike topical corticosteroids, sugarcane cellulosic polysaccharide gel presented desirable results for clinical practice, since this biomaterial was able to promote relief of symptoms without delaying tissue repair of induced oral traumatic ulcers in animals.

The effects observed in the EPS group were not superior to the negative control, however we must take into account that the animals did not have an underlying disease, such as diabetes mellitus or autoimmune disease that could compromise the inflammatory basis of repair. In this case, new studies comparing the healing of these lesions in animals with systemic inflammatory or immunological impairment would be important.

## Conclusion

These results suggest that topical application of EPS gel in TUs in animals promotes faster repair than triamcinolone ointment, without increasing the intensity of inflammatory infiltration under the lesion. Although the repair process was similar between the negative control group and the experimental group, the use of EPS gel was effective in controlling pain in animals, producing greater comfort in alimentation, and resulting in decreased weight loss, compared to animals which received no treatment. In addition, the medication did not irritate animal tissue, favoring the possibility of future studies in humans.

### Limitations

The animals were not monitored for longer than seven days, as during this period the negative control group already showed well-defined macroscopic healing. For studies with longer time for euthanasia, it would be necessary to use animals with comorbidities that affect inflammatory, immunological and defense pathways, such as diabetes mellitus or autoimmune disease, leading to longer healing times as a control group.

## Data Availability

All data sets were generated or analyzed in the current study.
